# Association between Exposure to the Chinese Famine in Different Stages of Early Life and Decline in Cognitive Functioning in Adulthood

**DOI:** 10.3389/fnbeh.2016.00146

**Published:** 2016-07-14

**Authors:** Chao Wang, Yu An, Huanling Yu, Lingli Feng, Quanri Liu, Yanhui Lu, Hui Wang, Rong Xiao

**Affiliations:** School of Public Health, Capital Medical UniversityBeijing, China

**Keywords:** Chinese famine, malnutrition, different life stages, cognitive functioning, early life

## Abstract

**Objective:** To investigate whether exposure to the Chinese Famine in different life stages of early life is associated with cognitive functioning decline in adulthood.

**Methods:** We recruited 1366 adults born between 1950 and 1964 and divided them into fetal-exposed, early childhood-exposed (1–3 years old during the famine), mid childhood-exposed (4–6 years old during the famine), late childhood-exposed (7–9 years old during the famine), and non-exposed groups. A selection of cognitive tests was administered to assess their cognitive performance. Association between malnutrition in different famine exposure periods and adult cognitive performance was estimated by multivariate logistic and multiple linear regression analyses.

**Results:** There were significant differences in cognitive performance between subjects exposed to famine during different life stages. For the general cognitive tests, fetal-exposed period was associated with decreased scores of the Mini-Mental State Examination (MMSE), and late childhood-exposed with decreased scores of the Montreal Cognitive Assessment (MoCA). We also found exposure to famine during mid and late childhood was associated with worse performance on the Stroop color and word test.

**Conclusion:** Famine exposure *in utero* and during childhood is associated with overall and specific cognitive decline, affecting selective attention and response inhibition particularly.

## Introduction

Cognitive functioning is thought to result from complex interactions between genetic and environmental exposures over the course of the life. Good nutrition and sufficient nutrients availability always links brain functioning and cognitive development (Guesry, 1998; Nyaradi et al., 2013). There is an intriguing hypothesis that specific environmental exposures at sensitive or critical time points can alter brain and cognitive development with life-long consequences (Hoeijmakers et al., 2014). Early undernutrition especially during pregnancy and several years after birth thus may be such an environmental exposure increasingly implicated in irreversible and long-term damage to the brain and behavioral function (Venables and Raine, 2012; Raikkonen et al., 2013; Waber et al., 2014b).

Scholars have showed a growing interest in assessing potential long-term cognitive and behavioral functioning consequences of early life nutritional deprivation and thus famines in human history provide distinct opportunities to undertake such research. Dutch famine at the end of World War II was an intense period of food shortage and had a 6-month well-defined but brief duration (de Rooij et al., 2006). Numerous epidemiological studies about the Dutch famine has focused on the impact of exposure during gestation on increased susceptibility to the development of psychiatric disorders and long-lasting cognitive functioning. Some have suggested that the risk of schizophrenia is increased among individuals conceived during the famine (Hoek et al., 1996) and simultaneously people exposed in early gestation had elevated risk of anti-social personality disorder (Neugebauer et al., 1999). Moreover, the association between prenatal famine exposure and affective psychoses (Brown et al., 1995) and depression (de Rooij et al., 2011) was also observed. Furthermore, the Dutch famine birth cohort study also suggested the cognitive functioning in adulthood may be adversely affected by periconceptional exposure to famine (Roseboom et al., 2011).

Compared with Dutch famine, the Chinese famine, whose most severe period persisted from 1959 to 1961, was of longer duration. Superimposed on widespread undernutrition and food unavailability, Chinese famine was regarded as one of the catastrophes in Chinese history (Mu and Zhang, 2011). Therefore, it was expected that the impact of Chinese famine on behavioral and cognitive development to be more marked than the Dutch famine. Similar study results of increased schizophrenia risk were also found in the Chinese famine (St et al., 2005; Song et al., 2009).

However, findings from studies on Dutch famine are contradictory, and they have mostly focused on prenatal exposure only (Stein, 2014). Besides, undernutrition in childhood has been increasingly implicated in adult physical and mental health, which indicated that the inconsistent results in the literature may be caused by different postnatal life exposures (Huang et al., 2010). Since the influence of undernutrition during pregnancy and/or in early postnatal life on cognitive functioning involving multiple domains in late adulthood has not been examined. The purpose of the current study was to estimate the association between them among the pre- and postnatally exposed Chinese famine survivors.

## Methods

### Study population

The data of this study derived from an ongoing epidemiological survey granted by the State Key Program of National Natural Science Foundation of China, which was aimed at investigating the role of dietary cholesterol on Alzheimer's disease (AD) and conducted in different regions from 2013 to 2018. The subjects were recruited from local large hospitals and screened with a series of cognitive functioning tests. The selection of the subjects was required to be satisfied a number of criteria, which included willingness to participate in the study, born between 1950 and 1964 years and Han Chinese residents. The exclusion criteria included suffering from cognitive decline or impairment caused by a history of cerebrovascular disease, depression, traumatic brain injury, drugs, and currently taking medication or dietary supplement to improve cognitive functioning. The study protocol was in accordance with the Declaration of Helsinki and ethically approved by the Ethics Committee of Capital Medical University (2013SY35). All participants were provided written informed consent at the beginning of the study.

### Famine age categories

The nationwide famine unexpectedly hit China in the late 1950s and continued to the early 1960s. The most severe period was from 1959 to 1961. We selected subjects born between October 1st, 1950, and September 30th, 1964 as our analytic population based on the study Li et al. (2010). Famine exposure is set up according to birth year and subjects were subsequently divided into five groups: non-exposed group (age = 51–53, birth year = 1962–1964, *n* = 237), fetal-exposed group (age = 54–56, birth year = 1959–1961, *n* = 217), early childhood-exposed group (age = 57–59, birth year = 1956–1958, 1–3 years old during the famine, *n* = 314), mid childhood-exposed group (age = 60–62, birth year = 1953–1955, 4–6 years old during the famine, *n* = 320), and late childhood-exposed group (age = 63–65, birth year = 1950–1952, 7–9 years old during the famine, *n* = 278). Our total sample size was 1366 subjects.

### Demographic and clinical assessment

The demographic and clinical characteristics of the subjects including age, gender, years of education, lifestyle, family history of dementia, and medical history were collected by face-to-face interviews at the baseline evaluation. Smoking status and alcohol consumption were also ascertained. We coded smoking and alcohol as current and other. Current smoking was defined as having smoked three or more cigarettes a week during the past 6 months before recruitment. Current alcohol consumption was defined as alcohol intake three or more times a week during the past 6 months before recruitment. Body mass index (BMI) was calculated as weight (kg)/height (m^2^).

### Cognitive assessment

A selection of well-established and conventional cognitive functioning tests on the basis of earlier research was used to assess respondents' cognitive performance, which took about 40 min to complete. All the interviews were done face to face in local hospitals by nurses or researcher who had attended unified training several times before. All the tests were carried out according to provided guidelines and procedures. The assessment contained the following cognitive functioning tests.

## The mini-mental state examination (MMSE)

The Chinese version MMSE was chosen for global cognitive status across multiple domains. As a rapid cognitive screening instrument and a practical method of grading cognitive functioning, it comprises 20 individual tests, totaling 30 points and covers 11 domains. The brevity and the broad coverage of cognitive domains make it the most commonly used cognitive instrument and diagnostic test of dementia (Mitchell et al., 2014). The cutoff score for dementia applied to the Chinese residents is 19 for illiterate individuals, 22 for individuals with 1–6 years of education and 26 for individuals with 7 or more years of education (Zhang et al., 1999).

## The montreal cognitive assessment (MoCA)

The Beijing version MoCA is also brief 30-point assessment of global cognitive screening instrument intended to detect mild cognitive impairment (MCI). It also provides a comprehensive assessment including a broad array of cognitive domains but incorporates expanded executive function and visuospatial items, which offers sensitivity and specificity to detect MCI patients and other cognitively impaired subjects with a normal range score on the MMSE (Gluhm et al., 2013; Lam et al., 2013). The cutoff score for MCI applied to the Chinese residents is 14 for illiterate individuals, 19 for individuals with 1–6 years of education, and 24 for individuals with 7 or more years of education (Lu et al., 2011).

## Logical memory test (LMT)

The test from the Wechsler Memory Scale-Revised, Chinese version (WMS-RC) edited by Gong et al. (1989) was adopted to evaluate memory functions in this study. Logical memory test (LMT) provided measures of verbal memory function and capacity to recall and acquire information over brief time periods. Participants tested by LMT were required to recall two story paragraphs told by investigators immediately. Gist and verbatim scoring systems were used to evaluate the verbal recall of the story paragraphs.

## The stroop color and word test (SCWT)

The test consists of three subtests: subtask I composed of names of four colors printed in black font (red, blue, yellow, and green), subtask II with patches in one of these colors and subtask III that consists of color names printed in an incongruous ink color. Each subtest displays 50 stimuli. Subjects were instructed to first read the color names (subtask I), then recognize color of the patches (subtask II), and finally name the ink color of the printed words (subtask III) as quickly as possible. The outcome of this test was completion time (in seconds) and correct number of each subtest. Stroop interference effects (SIE) were used as the analyzed index, which were composed of time for SIE, calculated according to the formula of (subtask III time- subtask II time), and correct number for SIE, calculated according to the formula of (subtask II correct number- subtask III correct number). The test assesses selective attention and processing speed as well as response inhibition, an index of executive function. Lower scores represent superior cognitive performance (Guo et al., 2005).

### Statistical analyses

The analysis was performed using IBM SPSS software (Version 19.0). All the analyses were two-sided and the statistically significant level was set at 0.05. Continuous variables were expressed as the mean ± standard deviation (SD) when normally distributed or medians (interquartile ranges, IQR) when non-normally distributed. And categorical values were shown in the form of frequencies (percentage,%). Univariate statistical analysis used the following tests: Pearson's chi-square test for categorical variables, analysis of variance (ANOVA), or the Mann–Whitney *U*-test for continuous variables, as appropriate. *Post-hoc* comparisons were evaluated using the Dunnett *t*-tests and Hochberg modification of the Bonferroni correction tests. To assess associations among life stages exposed to famine and cognitive functioning, multivariate logistic regression analyses, and multiple linear regression analyses were used. Non-exposed group was used as reference group. The model took into account potential confounders such as age, gender, years of education, lifestyle, and medical history and model parameters were estimated.

## Results

Table 1 shows the results of demographic and clinical characteristics by different life stages exposed to famine. Compared with non-exposed group, childhood-exposed subjects had significantly lower years of education.

**Table 1:** **Demographic and clinical characteristics of 1366 individuals exposed to the Chinese famine of 1959–1961**.

	**Childhood-exposed**			**Fetal-exposed**	**Non-exposed**
	**Late childhood**	**Mid childhood**	**Early childhood**		
N	278	320	314	217	237
Birth date					
(From October 1,year)	1950	1953	1956	1959	1962
(To September 30,year)	1952	1955	1958	1961	1964
Age in 2015	63–65	60–62	57–59	54–56	51–53
Women (%)	132 (47.5)	158 (49.4)	159 (50.6)	113 (52.1)	108 (45.6)
BMI (kg/m^2^)	25.36 ± 3.46	25.27 ± 3.23	25.00 ± 3.21	25.24 ± 3.70	25.72 ± 3.81
Years of education	9 (9, 12)^*^	9 (9, 12)^*^	12 (9, 12)^*^	12 (9, 12)	12 (9, 16)
Family history (%)	22 (7.9)	26 (8.1)	32 (10.2)	22 (10.1)	16 (6.8)
Current smoking (%)	78 (28.1)	79 (24.7)	82 (26.1)	61 (28.1)	76 (32.1)
Alcohol use (%)	76 (27.3)	85 (26.6)	100 (31.8)	71 (32.7)	80 (33.8)
Hypertension (%)	98 (35.3)	110 (34.4)	90 (28.7)	61 (28.1)	66 (27.8)
Hyperlipidemia (%)	56 (20.1)	67 (20.9)	65 (20.7)	46 (21.2)	35 (14.8)
Diabetes (%)	42 (15.1)	46 (14.4)	40 (12.7)	25 (11.5)	32 (13.5)
Coronary heart disease (%)	17 (6.1)	15 (4.7)	21 (6.7)	15 (6.9)	16 (6.8)

BMI, body mass index. Data are presented means ± SD, median (interquartile range), or n (%).

Data shown as median (interquartile range) were compared between 5 groups using the Mann–Whitney U-test.

Data shown as mean ± standard deviation were compared between 5 groups using the analysis of variance.

Data shown as n (%) were compared between 5 groups using the Pearson's chi-square test.

* Compare with non-exposed group using the Hochberg modification of the Bonferroni correction: the adjusted p for statistical significance was calculated as p' = p /[k (k – 1)/2] = 0.05/[5*(5 – 1)/2] = 0.005, p' < 0.005.

Table 2 presents the results of cognitive tests for each of the five groups. Subjects exposed to famine during any stage of childhood had significantly lower scores for MMSE and MoCA. Mid and late childhood exposed subjects had significantly longer time for SIE and only late childhood exposed subjects had lower correct for SIE than those with exposure in other periods. No substantive differences were observed among the groups on LMT.

**Table 2:** **Performance on tests of cognitive functioning among 1366 individuals**.

	**Childhood-exposed**			**Fetal-exposed**	**Non-exposed**
	**Late childhood**	**Mid childhood**	**Early childhood**		
MMSE	28 (27, 29.5)^*^	29 (27, 30)^*^	29 (27, 30)^*^	29 (27, 30)	29 (28, 30)
MoCA	25 (22, 27)^*^	25 (23, 27)^*^	25 (22, 27)^*^	26 (23, 27)	26 (24, 28)
Correct for SIE	1 (0, 4)^*^	0 (0, 3)	1 (0, 3)	1 (0, 3)	0 (0, 2)
Time for SIE	38 (29, 50)^*^	37 (28, 51)^*^	36 (27, 47)	35.5 (26, 45)	31 (25, 43)
LMT	10.70 ± 5.27	10.42 ± 5.11	10.37 ± 5.28	10.79 ± 5.48	11.03 ± 5.13

MMSE, Mini-Mental State Examination; MoCA, Montreal Cognitive Assessment; SIE. Stroop Interference Effects; LMT, Logical Memory Test.

Data are presented as means ± SD and median (interquartile range). Data shown as median (interquartile range) were compared between 5 groups using the Mann–Whitney U-test.

Data shown as mean ± standard deviation were compared between 5 groups using the analysis of variance.

Time for SIE was calculated according to the formula of (Stroop C time-Stroop B time).

Correct for SIE was calculated according to the formula of (Stroop B correct number-Stroop C correct number).

* Compare with non-exposed group using the Hochberg modification of the Bonferroni correction: the adjusted p for statistical significance was calculated as p' = p /[k (k – 1)/2] = 0.05/[5*(5 – 1)/2] = 0.005, p' < 0.005.

Table 3 shows no significant association of famine exposure with dementia screened by MMSE and MCI screened by MoCA in multivariate logistic analyses.

**Table 3:** **Association of exposure to the Chinese Famine in different life stages of early life with dementia screened by MMSE and MCI screened by MoCA from logistic regression analyses**.

	**Childhood-exposed**			**Fetal-exposed**	**Non-exposed**
	**Late childhood**	**Mid childhood**	**Early childhood**		
**DEMENTIA**					
N	3	6	12	9	4
OR (95%CI)^*^	3.23 (0.74–14.01)	1.68 (0.48–5.88)	2.01 (0.72–5.58)	1.96 (0.91–4.26)	Ref.
*p*	0.12	0.42	0.18	0.88	Ref.
**MCI**					
N	91	102	106	68	68
OR (95%CI)^*^	1.27 (0.43–3.76)	0.93 (0.37–2.34)	1.17 (0.55–2.48)	1.14 (0.64–2.03)	Ref.
*p*	0.66	0.88	0.68	0.66	Ref.

MCI, mild cognitive impairment; OR, odds ratio; Ref, reference group.

* Adjusted for demographic and clinical characteristics.

Table 4 provides the association of famine exposure with cognitive performance in multiple linear analyses. Exposure to famine during fetal period was associated with a 0.638 point decrease in the scores of MMSE and late childhood exposure was associated with a 0.680 point decrease in the scores of MoCA. Early childhood and late childhood exposure positively affected the correct for SIE. Meanwhile, mid childhood and late childhood exposure showed positive association with time for SIE. However, no association between worse performance on LMT and famine exposure was observed.

**Table 4:** **Association of exposure to the Chinese Famine in different life stages of early life with performance of cognitive functioning tests from multiple linear regression analyses**.

	**Childhood-exposed**			**Fetal-exposed**	**Non-exposed**
	**Late childhood**	**Mid childhood**	**Early childhood**		
**MMSE**					
Median (IQR)	28 (27, 30)	29 (27, 30)	29 (27, 30)	29 (27, 30)	29 (28, 30)
B	–0.336	–0.247	–0.358	–0.638	Ref.
*p*	0.112	0.227	0.078	0.004^*^	Ref.
**MoCA**					
Median (IQR)	25 (22, 27)	25 (23, 27)	25 (22, 27)	26 (23, 27)	26 (24, 28)
B	–0.680	–0.258	–0.542	–0.590	Ref.
*p*	0.037^*^	0.411	0.083	0.081	Ref.
**CORRECT FOR SIE**					
Median (IQR)	1 (0, 4)	0 (0, 3)	1 (0, 3)	1 (0, 3)	0 (0, 2.25)
B	1.292	0.061	0.749	0.422	Ref.
*p*	0.001^*^	0.872	0.048^*^	0.306	Ref.
**TIME FOR SIE**					
Median (IQR)	38 (29, 50)	37 (28, 51)	36 (27, 47)	36 (26, 45)	31 (25, 43)
B	5.942	7.261	4.249	2.625	Ref.
*p*	0.011^*^	0.002^*^	0.062	0.286	Ref.
**LMT**					
Mean ± SD	10.70 ± 5.27	10.42 ± 5.11	10.37 ± 5.28	10.79 ± 5.48	11.03 ± 5.13
B	0.778	0.397	0.017	–0.081	Ref.
*p*	0.119	0.410	0.971	0.875	Ref.

MMSE, Mini-Mental State Examination; MoCA, Montreal Cognitive Assessment; SIE, Stroop Interference Effects; LMT, Logical Memory Test; Ref, reference group.

Data are presented as means ± SD and median (interquartile range).

* Compare with non-exposed group, p < 0.05.

## Discussion

We investigated whether early-life malnutrition exposure *in utero* and during childhood to the Chinese famine would predict cognitive functioning in later life among Chinese born from 1950 to 1964. Although no association between exposure and general cognitive impairment including dementia screened by MMSE and MCI screened by MoCA was observed, exposure to famine *in utero* and during childhood is associated with cognitive decline in selective attention and response inhibition, as suggested by lower performance on Stroop color and word test. This study suggested intrauterine periods and a few years of postnatal life were critical for brain functioning and cognitive development.

The current study provides new and further evidence to the accumulating body of literature which have demonstrated early-life nutritional deprivation has a profound and long-term effect on cognitive development (Levitsky and Strupp, 1995; Galler et al., 2005, 2012; Lumey et al., 2011; Ampaabeng and Tan, 2013). The severe period of Chinese famine lasted 3 years, which allows an investigation of cognitive consequences of famine exposure in pregnancy and the first several years of life. We found no effects of pre- and postnatal famine exposure on cognitive impairment that meets screening criteria such as dementia and MCI, suggesting the effects were not severe enough to be linked with dementia and MCI. Despite that, we cannot exclude that exposure to famine is associated with slight changes and decline in cognitive functioning. As we can see from the results of multiple liner regression, exposure to famine during fetal period and late childhood was respectively associated with slightly decreased scores of MMSE and MoCA, indicating slight but significant impact of early life malnutrition on the cognitive development will persist into adulthood and result in a little poorer performance on general cognitive tests. Subsequently, subjects exposed during mid-childhood and late childhood performed significantly worse in time for SIE than non-exposed ones, providing evidence of the impairment of selective attention and response inhibition, an index of executive function. Stroop-like tests require selective attention and response inhibition (Van der Elst et al., 2006; Douris et al., 2015), which arises when subjects are completing the third conflicting part of the test between reading the words or naming the color of the words. Spending more time on choosing non-automatic response and inhibiting automatic response means poorer selective attention and response inhibition performance. Carter et al. (1997) have found the lower scores of color-incongruent Stroop task may be linked with less anterior cingulate gyrus activation among patients with schizophrenia. Moreover, age-related diffuse lesions of the white matter also have been reported to be associated with reduced Stroop tests and selective attention performance (van Swieten et al., 1996). Compared with Dutch famine studies, a Stroop color-word interference test found no impact of exposure to famine in the pregnancy on cognitive functioning (de Groot et al., 2011) whereas a Stroop-like task was associated with famine exposure *in utero* (de Rooij et al., 2010). The conflicting results of these studies demonstrate different association between maternal malnutrition during fetal life and performance of Stroop-like tasks. However, our results showed significant association with mid childhood and late childhood exposure. The inconsistent results may be due to various version and differently operationalized Stroop tests and different timing and duration of exposure.

Several cellular and molecular mechanisms might explain the different associations that fetal-exposed group has deficits in MMSE, late childhood-exposed group in MoCA and mid/late childhood-exposed group in Stroop color and word test. It should be noted that the prenatal period involves most of the process of neurogenesis. Perez-Garcia et al has demonstrated prenatal protein malnutrition in rats led to impaired encoding and consolidation of memory. This learning deficit was associated with reduced hippocampal neurogenesis (Perez-Garcia et al., 2016). However, in the postnatal period synaptogenesis takes place (Alamy and Bengelloun, 2012). Malnutrition during different phases can thus produce permanent alterations in different neurotransmitters systems, which may be responsible for different cognitive performance exhibited in MMSE, MoCA, and Stroop color and word test. It is also noteworthy that long-term intellectual compromise is associated with stunting in the first few years of life (Waber et al., 2014a). Nevertheless, a relevant study in Peru has showed that children who recovered from early stunting did not differ in cognitive functioning in contrast to non-stunted counterparts (Crookston et al., 2011). Since stunted children with subsequent catch-up growth or nutritional recovery had demonstrated normal levels of cognition, weight, and height, body compositions and bone mineral densities (Martins et al., 2011), the persisting effects of chronic postnatal exposure on brain need to be paid more attention and thus help previously malnourished children complete catch-up in mental and physical growth.

The effects of early-life famine exposure on cognitive development depend on a variety of factors (Huang et al., 2013), including timing and duration of exposure and severity of the famine as well as specific instruments and methods of grading cognitive functioning. Some potential threats to validity cannot be neglected. Possible confounders were not included such as birth state, regional disparity, and immigration (Wang et al., 2015). It may not be easy to judge which were really privileges among those confounders and the cognitive decline is not exclusively due to famine exposure, which may contribute to the seemingly small results of comparatively slight decrease in scores of tests.

Since the age at which malnutrition occurs is a critical factor, subjects exposed in different times of life also differs in age. The current study is an observational study with different age groups based on timing and duration of exposure being taken into analysis rather than different groups according to famine and non-famine exposure with comparable age, which mainly because almost all the areas in China were affected by the famine from 1959 to 1961, no valid subjects without famine exposure born at the same time was available. There is no doubt that cognitive functioning changes with normal human aging, and attention and memory are two basic cognitive functions that are most affected by aging. Therefore, the results of both multivariate logistic regression analysis and multiple linear regression analysis took into account potential confounders especially age and significant associations were still observed.

In a word, the study investigates famine exposure not only during gestation but also during infancy and childhood, which could be generalized to the effects of chronic undernutrition. However, it remains uncertain whether the results would be different if the study could establish Chinese Famine Birth Cohort and complete follow-up. Therefore, there are good reasons to plan in-depth study in the future and to evaluate relevant cognitive disorders such as neurodegenerative problems.

## Conclusion

The findings of the current study suggested that the cognitive decline ensuing from malnutrition during gestation and childhood caused by exposure to the Chinese Famine in different stages of early life is considerable. The impairment of selective attention and response inhibition was especially detected. Given the prevalence of malnutrition during gestation and childhood worldwide, the significant association between early-life malnutrition and long-term cognitive decline in our findings indicate that prevention of malnutrition in early life should remain a major public health goal. Meanwhile, early life interventions should be taken into consideration to help malnourished children to be fully rehabilitated nutritionally and complete catch-up in mental and physical growth, thus mitigating these significant neurodevelopmental insults.

## Author contributions

RX designed experiments; CW, YA, HY, YL, HW, LF, and QL carried out experiments; CW and YA analyzed experimental results and wrote the manuscript.

## Funding

The research was supported by the State Key Program of the National Natural Science Foundation of China (Grant No. 81330065).

### Conflict of interest statement

The authors declare that the research was conducted in the absence of any commercial or financial relationships that could be construed as a potential conflict of interest.
